# The Congress Museum

**Published:** 1901-07-27

**Authors:** 


### The Congress Museum.

The Congress Museum, although not a great one, was full of interesting material. As showing how true the tubercle-bacillus breeds, and how definite an entity it is, the first object mentioned in the catalogue is a collection of culture tubes containing pure cultures of the tubercle-bacillus inoculated on June 20th, 1901, which are the direct descendants of a culture taken on August 15th, 1881, just 20 years ago, from a case of miliary tuberculosis. Since that date it has undergone 435 sub-cultures without again being passed through the body of an animal. This exhibit is a little thing, which to the uninitiated may appear trivial and unmeaning. But it demonstrates a fact of the greatest moment, namely, that in this bacillus we do not have to do with an accidental product due to a particular environment, but with a distinct living form as definite as cat or dog, or, indeed, as man himself.

Further on we came to another still smaller exhibit, but of, perhaps, still greater interest, namely, three microphotographs, shown by Dr. Arthur Ransome, F.R.S., of cultures of the bacillus of tubercle (human) obtained by cultivating at ordinary temperatures, upon sterilised paper, moistened with condensed aqueous vapour from human breath and from ground air. The bacilli here represented have certain peculiarities no doubt showing, what we know to be the case with many microphytes, a tendency to assume abnormal forms under abnormal conditions, but showing also, what is the important point, that the bacillus can maintain its existence outside the human body, not only when petted and coddled on a certain medium, which by much experimentation has been found suitable for its growth, but when cultivated on paper. This little exhibit is chiefly valuable by its suggestiveness, pointing as it does to the likelihood of the bacillus growing as a saprophyte on walls and wall papers and rendering the house itself an abiding source of infection to generation after generation of sufferers from phthisis. Then we find the really great exhibit of the museum, namely, an endless series of pathological specimens showing the many forms which tuberculosis of the various organs may assume in the human body, including many microscopic slides, an interesting series of skiagrams by Dr. Hugh Walsham, and a series of casts, exhibited by Dr. J. J. Galbraith, showing the progressive change in the shape of the fingers and nails met with in phthisis pulmonalis.

To Londoners, at any rate, one of the most interesting exhibits was that by Mr. Shirley Murphy, medical officer to the County of London, which by aid of charts and diagrams put into striking form the relation of mortality from tuberculous diseases to overcrowding. There were also many statistical tables and diagrams, and a large number of plans, tables, etc., relating to hospitals and sanatoria.

